# Xiaoyaosan Decoction, a Traditional Chinese Medicine, Inhibits Oxidative-Stress-Induced Hippocampus Neuron Apoptosis In Vitro

**DOI:** 10.1155/2012/489254

**Published:** 2012-01-29

**Authors:** Zhen-zhi Meng, Jing-hong Hu, Jia-xu Chen, Guang-xin Yue

**Affiliations:** ^1^School of Preclinical Medicine, Beijing University of Chinese Medicine, Beijing 100029, China; ^2^Institute of Basic Theory in Chinese Medicine, Chinese Academy of Chinese Medical Sciences, Beijing 100700, China

## Abstract

Xiaoyaosan (XYS) decoction is a famous prescription for the treatment of mental disorders in China. In this experiment, we explored the way in which XYS decoction-reverse hippocampus neuron apoptosis in vitro. We used XYS decoction-containing serum to treat oxidative-stress-induced hippocampus neuron apoptosis and used immunofluorescence to determine the concentration of free calcium, mitochondrial membrane potential, and apoptotic rate of neuron. Results showed that 3-hour oxidative stress decrease mitochondrial membrane potential, increase the concentration of free calcium and apoptotic rate of neuron via triggering pathological changes of nucleus such as karyorrhexis, karyopyknosis. Low, medium, high dose of XYS-decoction-containing serum could reverse these phenomenon, and the effect of low-dose XYS-decoction-containing serum was significant in improving mitochondrial membrane potential and apoptotic rate of neuron. These findings suggest that XYS decoction may be helpful in reducing oxidative-stress-induced hippocampus neuron apoptosis.

## 1. Introduction

The neuronal system plays a leading role in reaction to stress. Chronic stress has close relationship with depression. Hippocampus is the target for the stress hormone and the hippocampus neuron may be the material basis to trigger depression. The structure and function of hippocampus neuron will be damaged by chronic stress not by acute stress. 

Traditional Chinese medicine (TCM) has an active effect on chronic disease and psychiatry. XYS decoction created in *Song* Dynasty (960-1127 AD) contains Radix Angelicae Sinensis, Poria, Radix Paeoniae Alba, Radix Glycyrrhizae, Radix Bupleuri, Rhizoma Atractylodis Macrocephalae, Herba Menthae, and Rhizoma Zingiberis Recens. The chemical constituent of XYS includes peoniflorin, saikoside, ferulic acid, atractylol, glycyrrhetate, curcumin, and menthone [1]. XYS decoction has been mainly used to treat liver stagnation and spleen deficiency syndrome (LSSDS) and mental disorders in TCM clinic. The function of XYS decoction is to soothe the liver, improve the circulation of qi, relieve depression, strengthen the spleen, and nourish blood. It is a safe and useful prescription in clinical. Our previous studies showed that XYS decoction on the treatment of patients with LSSDS may be through enhancing plasma *β*-EP and decreasing E and DA release [2], and the therapeutic antidepression actions are due to protection of brain neurotrophin factor [3]. 

The objective of this study was to observe the effect of different doses of XYS decoction on the hippocampus neuron via oxidative-stress-induced hippocampus neuron apoptosis. Chinese composite recipe aims at multitarget in human body and includes many different materials and chemical compositions. After Chinese composite recipe is taken, metabolism will happen in stomach, intestine, and liver and the effect of Chinese composite recipe will play a role [4]. Therefore, to reproduce the features of XYS after metabolism in digestive system, we prepared XYS-containing serum. We hope that this experiment can provide experimental evidence about XYS decoction's function in reversing hippocampus neuron apoptosis in rats induced by oxidative stress.

## 2. Materials and Method

### 2.1. Animal

12 male and 12 female Sprague-Dawley rats (Beijing Weitong Lihua Research Center for Experimental Animals), weighing 216–380 g, were used in the experiments. Animals were housed in a room with routine care (20–24°C, relatively humidity of 30–40%) and free access to food and water. This study was performed in strict accordance with the recommendations in the Guide for the Care and Use of Laboratory Animals of China. The protocol was approved by the Committee on the Ethics of Animal Experiments of the Beijing University of Chinese Medicine (BUCM). All surgery was performed under sodium pentobarbital anesthesia, and every effort was made to minimize suffering.

### 2.2. Preparation of Extracts of the XYS Decoction

The XYS decoction consists of the following dried raw materials: 150 g of *Poria cocos* (Schw.) Wolf (Poria), 300 g of *Paeonia lactiflora* Pall. (Radix Paeoniae Alba), 150 g of *Glycyrrhiza uralensis* Fisch. (Radix Glycyrrhizae), 300 g of *Bupleurum chinense* DC. (Radix Bupleuri), 300 g of *Angelica sinensis* (Oliv.) Diels (Radix Angelicae Sinensis), 300 g of *Atractylodes macrocephala* Koidz. (Rhizoma Atractylodis acrocephalae), 100 g of *Mentha haplocalyx *Briq. (Herba Menthae), and 100 g of *Zingiber officinale* Rosc. (Rhizoma Zingiberis ecens). These eight herbs were purchased from Medicinal Materials Company of Beijing Tongrentang, processed Sino-Japan Friendship Hospital (Beijing) abiding by *Regulation on Processing of Traditional Chinese Medical Herbal Pieces of Beijing*.

### 2.3. Preparation of Xiaoyaosan-Containing Serum

References [5–9] 24 SD rats were randomly divided into four groups: (1) high-dose XYS decoction containing serum, (2) moderate-dose XYS-decoction-containing serum, (3) low-dose XYS-decoction-containing serum, and (4) vehicle control. The first 3 groups were gavaged with intragastric XYS decoction twice daily for 7 consecutive days (7.708 g/kg/d, 3.854 g/kg/d, 1.927 g/kg/d for high, moderate, low dose resp.), the last group was gavaged with intragastric deionized water. XYS decoction for rats was diluted by deionized water. 1 mL/100 g body weight XYS decoction for intragastric. Blood was collected 1 hour after the last administration via abdominal aorta and then centrifuged. Serum of the same group was pooled, filtered through 0.22 *μ*mol/L filter, and inactivated at 56°C for 30 minutes, split and stored at –20°C.

### 2.4. Cell Culture and Growing

Hippocampuses were harvested from SD rats born in 24 hours. Hippocampal neurons were planted into 24 wells after digestion with trypsin. The first day DMEM/F12 and fetal bovine serum were added into the plate. After that Neurobasal, B27 and L-Glutamine took the place of DMEM/F12 and fetal bovine serum for 6 days. In this period, totally new Neurobasal + B27 + L-Glutamine would be added every 3 days and used ones would be discarded. On the 7th day, hippocampal cells of rats are in good condition for following experiments [10]. We tested the viability with trypan blue and purity with MAP-2. Once the viability and purity reach 90%, subsequent experiments would follow up. Neurons were divided into 6 groups, that is, control group, oxidative stress group, vehicle control group, oxidative stress + high-dose XYS-decoction-containing serum group, oxidative stress + moderate-dose XYS-decoction-containing serum group, oxidative stress + low-dose XYS-decoction-containing serum group. (To make sure the difference of effects of XYS-decoction-containing serum is from XYS not from serum, vehicle control is needed. Oxidative stress group was exposed to Neurobasal + B27 + L-Glutamine after oxidative stress, while vehicle control group was exposed to serum only.) All the groups except the control group were treated with 1 mmol/L H_2_O_2_ for 1 hour [11]. After that we discarded nutrient fluid with H_2_O_2_ and gave 10% XYS-decoction-containing serum or vehicle (the serum) to cultivate for 72 hours. After that we gave different treatments to test mitochondrial membrane potential, concentration of free calcium, and apoptotic rate of neuron.

### 2.5. Concentration of Free Calcium Assay

Cells stained with Fluo3/AM (0.5 *μ*M) were incubated in 37°C for 60 minutes. Cells were washed with D-Hanks for 3 times (5 min/time). Incubation in 37°C again is needed before being monitored with fluorescent microscope. Excitation wavelength is 488 nm.

### 2.6. Apoptotic Rate of Neuron Assay

Cells were fixed with 4% paraformaldehyde for 10 minutes. Hoechst 33258 (1 mg/mL) was used to stain cells at room temperature in dark for 5 min. Cells were washed twice with PBS and examined and immediately photographed under a fluorescence microscope with an excitation wavelength of 330–380 nm. Apoptotic cells were defined on the basis of nuclear morphology changes such as karyorrhexis, karyopyknosis.

### 2.7. Mitochondrial Membrane Potentials Assay

After treatments, cells were incubated with an equal volume of JC-1 (5, 5′, 6, 6′-Tetrachloro-1, 1′, 3, 3′-tetraethyl-imidacarbocyanine iodide) staining solution (10 *μ*g/mL) at 37°C for 20 min and rinsed twice with PBS. Mitochondrial membrane potentials were monitored by determining the relative amounts of dual emissions from mitochondrial JC-1 monomers or aggregates using a laser confocal microscope (ZEISS LSM510 META), 488 laser excitation for green and 540 excitation emission for red. The ratios of red/green fluorescent densities were calculated.

### 2.8. Statistical Method

Data are expressed as mean ± SEM calculated from the test groups. The analysis of variance (ANOVA) was performed for comparison among groups. Significance was accepted at the *P* < 0.05 level.

## 3. Result

### 3.1. Effect of XYS Decoction on Cell Viability

**Figure 1:**
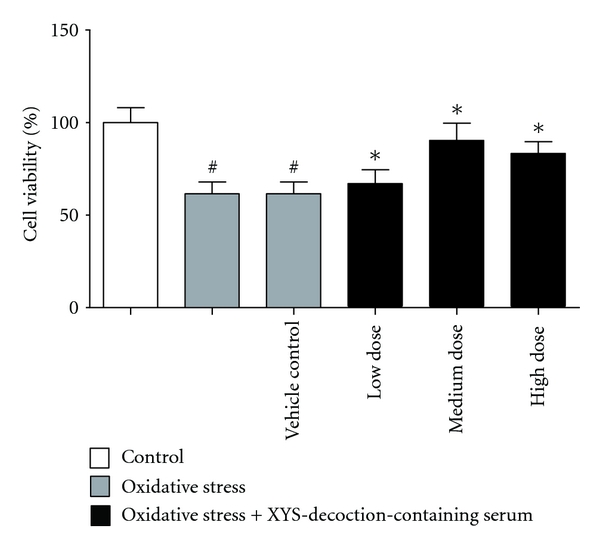
The effect of XYS decoction on cell viability. Each value represents mean ± SEM. Oxidative stress group and vehicle control group showed significant decrease in comparison with the control group in the cell viability, *P* < 0.05. Oxidative stress plus different doses of XYS-decoction-containing serum groups showed significant increase in comparison with oxidative stress group and vehicle control group in cell viability, *P* < 0.05.

In this test, the cell viability of oxidative stress group and vehicle control group was lower than control group, *P* < 0.05. The cell viability of oxidative stress group + XYS-decoction-containing serum group was higher than oxidative stress group and vehicle control group, *P* < 0.05 (^#^ *P* < 0.05 versus control group, *P < 0.05 versus oxidative stress group and vehicle control group) (Figure 1).

### 3.2. Effect of XYS Decoction on Concentration of Free Calcium

**Figure 2:**
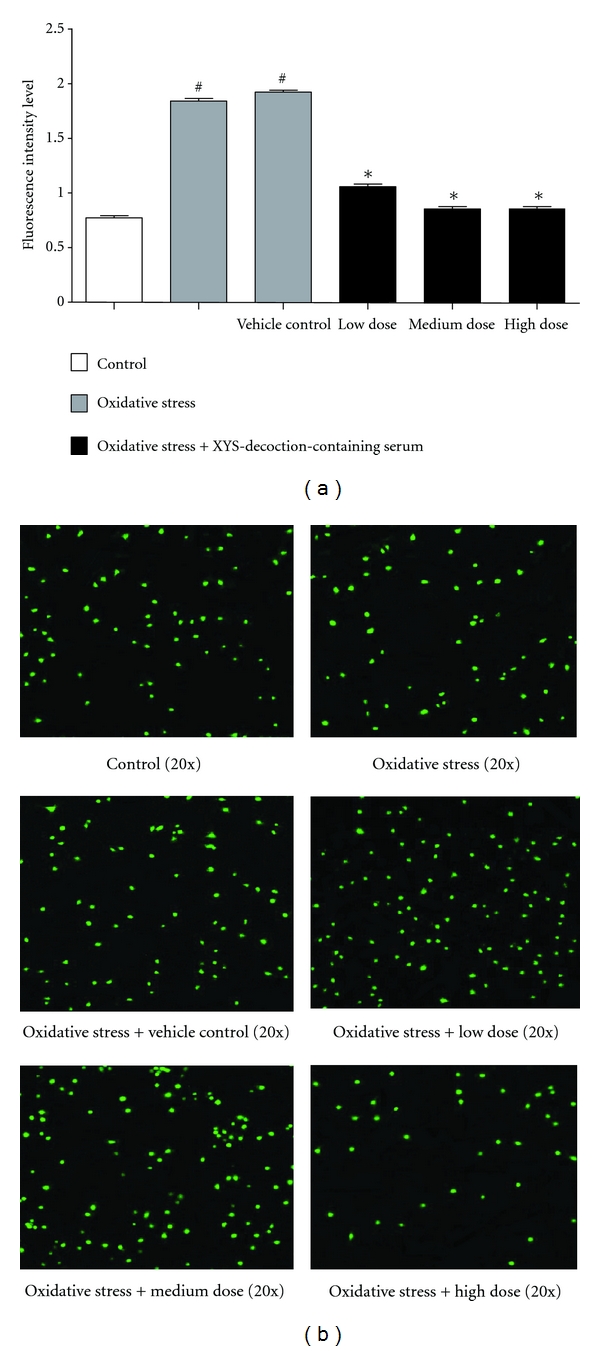
The effect of XYS decoction on concentration of free calcium. (a) Each value represents mean ± SEM. Oxidative stress group and vehicle control group showed significant increase in comparison with the control group in the concentration of free calcium, *P* < 0.05. Oxidative stress plus different doses of XYS-decoction-containing serum groups showed significant decrease in comparison with oxidative stress group and vehicle control group in the concentration of free calcium, *P* < 0.05. (b) Concentration of free calcium was measured by fluorescent microscope with Fluo3/AM. Fluorescence intensive value of green spots was measured to indicate concentration of free calcium.

In this test, concentration of free calcium in oxidative stress group and vehicle control group was higher than that in control group significantly, *P* < 0.05. Concentration of free calcium in oxidative stress plus different doses of XYS-decoction-containing serum groups was lower than that in oxidative stress group and vehicle control group significantly, *P* < 0.05 (^#^ *P* < 0.05 versus control group, *P < 0.05 versus oxidative stress group and vehicle control group) (Figure 2(a)). Concentration of free calcium was measured by fluorescent microscope with Fluo3/AM (Figure 2(b)).

### 3.3. Effect of XYS Decoction on Apoptosis Rate of Neuron

**Figure 3:**
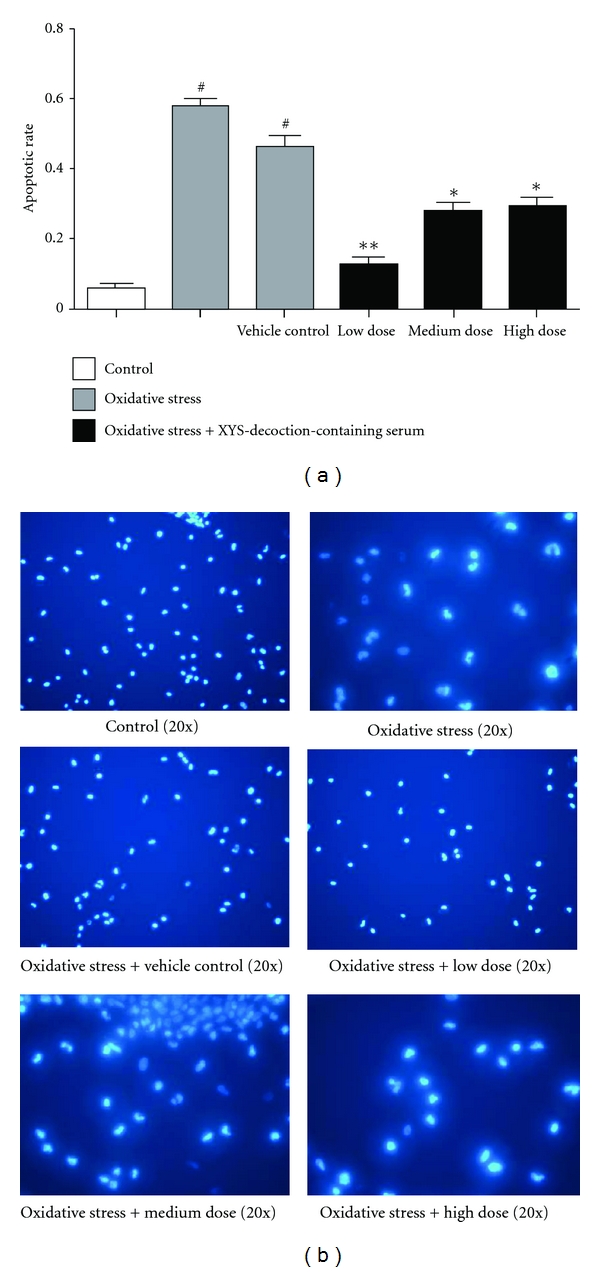
The effect of XYS decoction on apoptotic rate of neuron. (a) Each value represents mean ± SEM. Apoptotic rate of neuron of oxidative stress group and vehicle control group was higher than that in control group significantly, *P* < 0.05. Apoptotic rate of neuron in oxidative stress + medium-, high-dose XYS-decoction-containing serum groups was lower than that in oxidative stress group and vehicle control group significantly, and oxidative stress + low-dose XYS-decoction-containing serum group was lower than that in oxidative stress group and vehicle control group significantly. (b) Morphological changes of neurons were captured by fluorescent microscope with Hoechst 33258. The ratio equals the number of the karyokinesis divided by the number of total neurons in same fields.

In this test, apoptotic rate of neuron in oxidative stress group and vehicle control group was higher than that in control group significantly, *P* < 0.05. Apoptotic rate of neuron in oxidative stress + medium-, high-dose XYS-decoction-containing serum groups was lower than that in oxidative stress group and vehicle control group significantly, *P* < 0.05; and oxidative stress + low-dose XYS-decoction-containing serum group was lower than that in oxidative stress group and vehicle control group very significantly, *P* < 0.01. (^#^ *P* < 0.05 versus control,*P < 0.05 versus oxidative stress group and vehicle control group, **P < 0.01 versus oxidative stress group and vehicle control group) (Figure 3(a)). Representative images (20x) were scanned by fluorescent microscope with Hoechst 33258. The ratios equal the number of the karyokinesis divided by the number of total neurons in this field (Figure 3(b)).

### 3.4. Effect of XYS Decoction on Mitochondrial Membrane Potential

**Figure 4:**
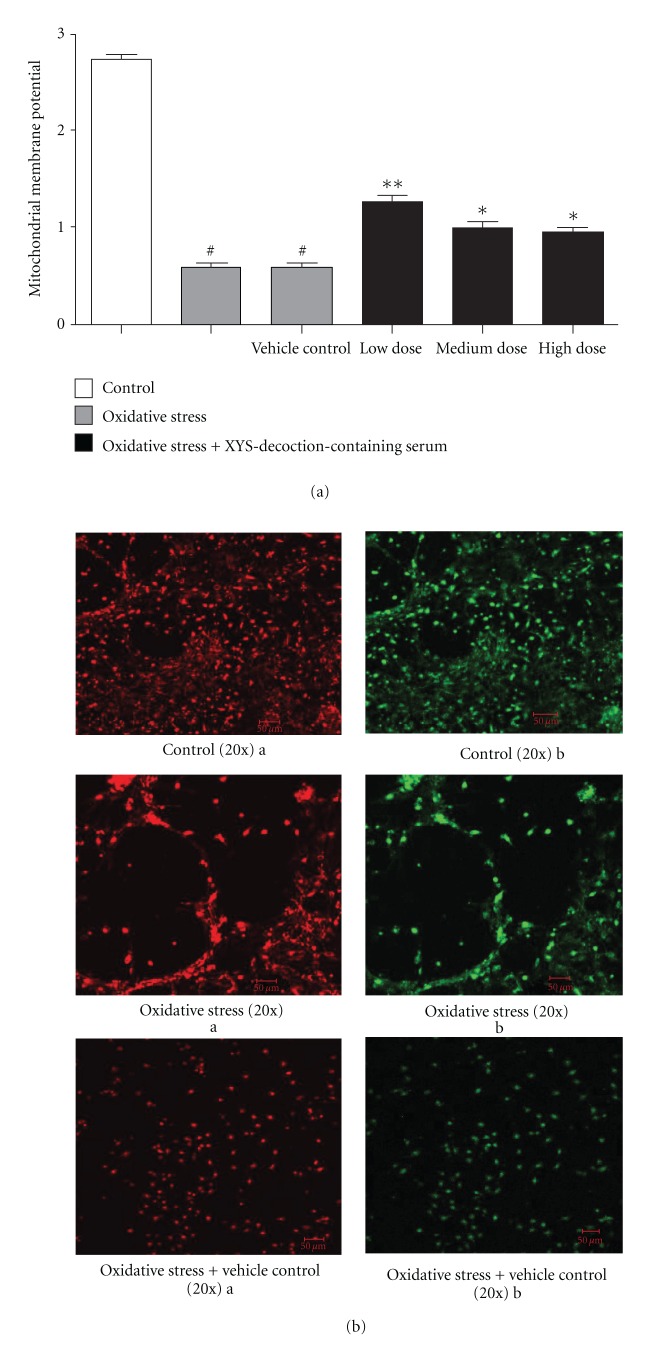

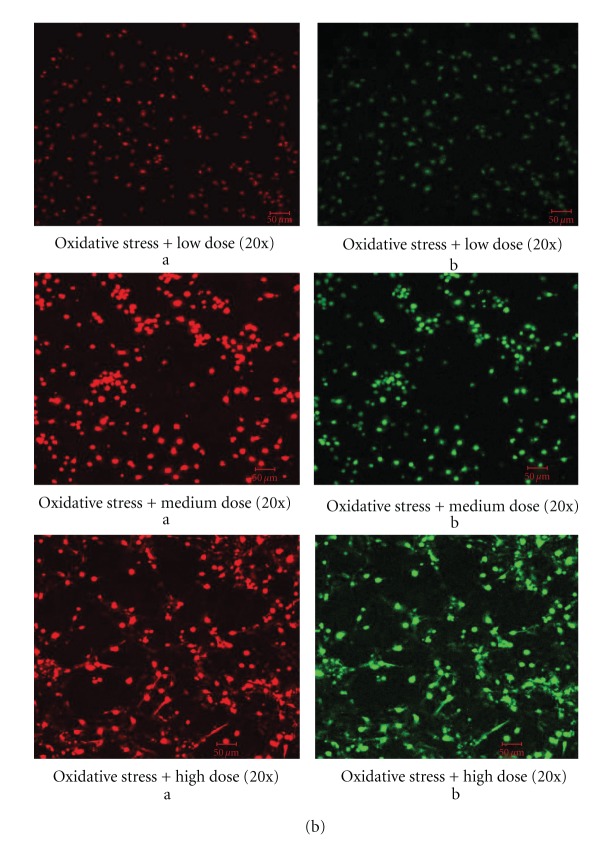
The effect of XYS decoction on mitochondrial membrane potential. (a) Each value represents mean ± SEM. Mitochondrial membrane potential in oxidative stress group and vehicle control group decreased significantly compared with control group. Mitochondrial membrane potential in oxidative stress plus different doses of XYS-decoction-containing serum groups increased significantly compared with oxidative stress group and vehicle control group. Furthermore, the function of oxidative stress + low-dose XYS-decoction-containing serum group was stronger compared to oxidative stress + medium-, high-dose XYS-decoction-containing serum groups. (b) Representative images were scanned by a confocal microscope after staining with JC-1. The mitochondrial membrane potential was represented with ratio of red/green fluorescent densities.

In this test, mitochondrial membrane potential in oxidative stress group and vehicle control group was lower than that in control group significantly, *P* < 0.05. Mitochondrial membrane potential in oxidative stress plus different doses of XYS-decoction-containing serum groups was higher than that in oxidative stress group and vehicle control group significantly, *P* < 0.05; and the function of oxidative stress + low-dose XYS-decoction-containing serum group was stronger compared to the oxidative stress + medium-, high-dose XYS-decoction-containing serum groups, *P* < 0.01 (^#^ *P* < 0.05 versus control, *P < 0.05 versus oxidative stress group and vehicle control group, **P < 0.01 versus oxidative stress group and vehicle control group) (Figure 4(a)). Representative images (20x) were scanned by a confocal microscope after staining with JC-1 (Figure 4(b)). 

## 4. Discussion

Stress can impair the function of hippocampus. However, we know little about the subtle mechanism. Experiment showed that damage of hippocampus structure has relationship with its neuron apoptosis [12]. Mitochondria play a key role in apoptosis. Mitochondrial membrane potential is the bridge between mitochondria and apoptosis. Decreasing of the mitochondrial membrane potential is the beginning of the cascade and it is irreversible [13–17]. Apoptosis of the mitochondrial membrane potential is an important part in neuron apoptosis.

Mitochondria is the biggest calcium pool in the cell and it is very sensitive to calcium signal. Concentration of free calcium increases while cell apoptosis appears [18]. Free calcium in cell served as second messenger is one of the signal transduction pathways [19–21]. There is a difference between concentration of calcium in the cell (100 nmol/L) and out of the cell (1 mmol/L). Abnormity of the concentration of calcium in the cell will lead to abnormal neuronal excitability. Previous results showed that XYS-decoction-containing serum can restrain calcium overload and low mitochondrial membrane potential to inhibit corticosterone-induced apoptosis in PC12 cells to counter depression [22]. In addition, karyopyknosis, cytoplasm concentration, cell shrinking, and frame of the cell disorganization are the morphological characteristics of apoptosis. Morphological changes in the nuclear are remarkable. 

Therefore, the above data suggested that XYS decoction can regulate oxidative-stress-induced hippocampus neurons in mitochondrial membrane potential, concentration of free calcium and apoptotic rate of neuron. Low-dose XYS decoction seems better than medium-, high-dose XYS decoction in regulating mitochondrial membrane potential and apoptotic rate of neuron. These findings suggest that XYS decoction may decrease oxidative-stress-induced hippocampus neuron apoptosis and may help to elucidate its therapeutic antidepression actions are due to protection of hippocampus neurons and would provide scientific evidence for using this formula in clinic.
